# Structure and Transport Properties of Dense Polycrystalline Clathrate-II (K,Ba)_16_(Ga,Sn)_136_ Synthesized by a New Approach Employing SPS

**DOI:** 10.3390/ma9090732

**Published:** 2016-08-26

**Authors:** Kaya Wei, Xiaoyu Zeng, Terry M. Tritt, Artem R. Khabibullin, Lilia M. Woods, George S. Nolas

**Affiliations:** 1 Department of Physics, University of South Florida, Tampa, FL 33620, USA; kayawei@mail.usf.edu (K.W.); artem1@mail.usf.edu (A.R.K.); lmwoods@usf.edu (L.M.W.); 2 Department of Physics and Astronomy, Clemson University, Clemson, SC 29634, USA; xzeng@g.clemson.edu (X.Z.); ttritt@clemson.edu (T.M.T.)

**Keywords:** clathrate, SPS processing, thermoelectrics

## Abstract

Tin clathrate-II framework-substituted compositions are of current interest as potential thermoelectric materials for medium-temperature applications. A review of the literature reveals different compositions reported with varying physical properties, which depend strongly on the exact composition as well as the processing conditions. We therefore initiated an approach whereby single crystals of two different (K,Ba)_16_(Ga,Sn)_136_ compositions were first obtained, followed by grinding of the crystals into fine powder for low temperature spark plasma sintering consolidation into dense polycrystalline solids and subsequent high temperature transport measurements. Powder X-ray refinement results indicate that the hexakaidecahedra are empty, K and Ba occupying only the decahedra. Their electrical properties depend on composition and have very low thermal conductivities. The structural and transport properties of these materials are compared to that of other Sn clathrate-II compositions.

## 1. Introduction

Inorganic clathrates are a class of materials that have frameworks built up from group 14 atoms, and have been known since the work of Cros and co-workers [1,2]. They have crystal structures closely related to those of clathrate hydrates [3]. The group 14 atoms typically have tetrahedral coordination that form a network encapsulating “guest” species. Most of the research thus far has focused on compositions with the clathrate-I crystal structure, in part due to their interest for thermoelectric applications, although recently there has been a focus on developing new processing techniques for the synthesis of clathrate-II materials [4,5,6,7,8,9,10]. The clathrate-I crystal structure can be represented by the general formula X_2_Y_6_E_46_, and that of the clathrate-II crystal structure by the general formula X_8_Y_16_E_136_, where X and Y are encapsulated guest atoms in the two different interstitial sites, and E represents the group 14 element Si, Ge, or Sn. It is sometimes instructive to think of these structures as being constructed from two different face-sharing polyhedra; sixteen dodecahedra (E_20_) and eight hexakaidecahedra (E_28_) for the clathrate-II crystal structure, for example. Many different compounds are possible within these structure types [3].

Inorganic clathrates are of fundamental interest from the perspective of both bonding and their physical properties [11,12,13,14,15,16,17,18,19,20,21,22]. The transport properties of several compounds with the clathrate-I crystal structure have been investigated, but much less has been reported on the transport properties of clathrate-II compositions [3,12,14,15]. In the case of Sn clathrates, this discrepancy is even more glaring. Although Sn compositions with the clathrate-I structure have been studied over the past two decades [23,24,25,26,27,28,29,30,31], clathrate-II Sn materials have only recently been investigated, primarily for thermoelectric applications, although Kröner et al. [32] first reported the synthesis of Ba_16_Ga_32_Sn_96_—Ba being the guest atom, while Ga and Sn form the framework—almost two decades ago. Mano et al. [18] reported low temperature transport on single crystals of K_8+*x*_Ba_16−*x*_Ga_40−*y*_Sn_96−*z*_[ ]*_y_*_+*z*_, in effect reporting that the K resides inside both polyhedra, Ba resides preferentially on the hexakaidecahedra and vacancies exist on the framework. Koda et al. [19] reported the above room temperature thermoelectric properties of polycrystalline K_7.1_Ba_16.9_Ga_41.3_Sn_94.7_ synthesized by reaction of the elements. The authors of that paper specifically noted that no vacancies exist on the framework or the interstitial sites. They later reported on the optimization of the thermoelectric properties by adjusting the carrier concentrations [20]. Shäfer and Bobev [21,22] reported single crystal synthesis and structural properties of different Sn clathrate-II compositions, and indicated that K_2_Ba_14_Ga_30.4_Sn_105.6_ had no vacancies on the framework sites, with no alkali or alkali-earth occupancy in the hexakaidecahedra. No transport properties were shown in that study, due to the fact that the crystals were very small. Although different synthetic approaches can result in different relative compositions and different probabilities of defect formation, the stark differences in the experimentally-determined compositions for the Sn clathrate-II materials in these reports is noteworthy. In addition, one would expect single-crystal formation from similar self-flux methods to yield similar compositions. In the study reported herein, we grew small single crystals of K- and Ba-containing Sn clathrate-II compositions, with Ga substitution on the Sn sites, and subsequently ground the crystals into fine powder for spark plasma sintering (SPS) densification into dense polycrystalline materials for high temperature transport properties measurements, in order to present their physical properties and attempt to elucidate the differences in the reported data on these materials.

## 2. Sample Preparation

Potassium chunk (99.99%, Alfa Aesar, Ward Hill, MA, USA), Ba shot (99.9999%, Alfa Aesar), Ga pellets (99.99999%, Alfa Aesar), and Sn powder (99.999%, Alfa Aesar), in a 1:2:5.875:12 atomic ratio, were loaded into a tungsten crucible that was then sealed inside a custom-designed stainless steel vessel, with all synthetic procedures occurring inside a nitrogen glovebox. The stainless steel vessel was then transferred into a glass tube and sealed under vacuum. The glass tube was put into a furnace at 923 K for 15 h, followed by slow cooling to 723 K at a rate of one degree per minute before being air cooled to room temperature. The products of this reaction included clathrate-II (K,Ba)_16_(Ga,Sn)_136_, clathrate-I (K,Ba)_8_(Ga,Sn)_46_, BaSn_3_, Ba_5_Sn_3_, Ba_2_Sn, as well as elemental Sn and Ga. A mixture of 10 mL HCl, 10 mL HNO_3_, and 80 mL DI water was used to remove this remaining flux from the resulting products. Clathrate-II single-crystals had a specific octahedral shape, unlike the other products of the reaction (e.g., square-shaped clathrate-I crystals), and were separated manually for further processing, which will be described below. Single-crystal X-ray diffraction (XRD) for the two measured clathrate-II crystals indicated no “guest” atoms on the (Ga,Sn)_28_ hexakaidecahedra. This synthesis procedure was repeated in order to obtain enough clathrate-II single crystals for the next step in the process. In order to investigate the ability to vary the composition of the product, as well as to obtain a second clathrate-II composition for the investigation of transport properties, we reproduced the above procedure with a K:Ba:Ga:Sn atomic ratio of 1:2.5:6.75:13.75. The resulting products were very similar; however, the refinement results indicated a smaller K-to-Ba ratio than that of the previous specimen.

The small clathrate-II crystals from the two different batches were ground to fine powder and sieved (400 mesh) for SPS densification into two different polycrystalline specimens. Several batches of crystals were “sacrificed” in order to optimize the SPS densification process, so as to specifically determine the best densification parameters—the parameters that would result in the highest density with no impurities after SPS. One-half inch inner diameter graphite tooling, with graphite foil separating the powder and the punches and die, was used. Approximately 1.5 g powder was used for densification of each specimen. The parameters were 400 MPa and 483 K for 10 min, with a current pulse ratio of 20:5 ms, which resulted in phase-pure polycrystalline bulk specimens with a relative density of 90% as determined by measuring the dimensions and mass of the pellets after SPS. Powder XRD (PXRD) and electron probe analyses (EDX) were used to examine the purity and chemical composition of the densified specimens. EDX of the pellets was accomplished with an Oxford INCA X-Sight 7582M-equipped SEM (JEOL JSM-6390LV, High Wycombe, UK). The average atomic ratios were calculated from at least twelve data sets obtained from random positions of the pellet for each specimen, and indicated homogenous polycrystalline pellets with EDX compositions that corroborated the PXRD refinement results (Table 1). Before and after densification, PXRD data were collected with a Bruker D8 Advance diffractometer (Karlsruhe, Germany) equipped with a Lynxeye detector using Cu K_α_ radiation. Structure refinements were accomplished by the Rietveld method using the GSAS suite of programs [33,34]. Single crystal XRD data were collected using a Bruker AXS SMART APEX II CCD diffractometer (Madison, WI, USA) using Cu K_α_ radiation (*λ* = 1.54178 Å).

High temperature Seebeck (*S*) and resistivity (*ρ*) were measured on parallelepipeds (2 mm × 2 mm × 10 mm), cut from the pellets using a wire saw, with an ULVAC ZEM-3 system (Boston, MA, USA, experimental uncertainty of 5%–8% for *S* and *ρ* at elevated temperatures). High temperature thermal conductivity (*κ*) values were determined using the equation *κ* = *DαC*_p_. Thermal diffusivity measurements employed the laser flash method in a flowing He environment with a NETZSCH LFA 457 system (Selb, Germany). The uncertainty in the thermal diffusivity measurements were ~5%. Heat capacity *C*_p_ (≈*C*_v_) was estimated by the Dulong–Petit limit (*C*_v_ = 3*nR*, where *n* is the number of atoms per formula unit and *R* is the ideal gas constant). At high temperature, this may result in an underestimate of *C*_p_, thus affecting *κ*. However, it is a relatively good method for comparing the effect of doping and small compositional changes, since it eliminates the uncertainties associated with *C*_p_ measurements [35]. Room temperature Hall measurements were conducted on 0.5 × 2 × 5 mm^3^ parallelepipeds at multiple positive and negative magnetic fields in order to mitigate voltage probe misalignment effects (5% uncertainty).

## 3. Results and Discussion

**Figure 1:**
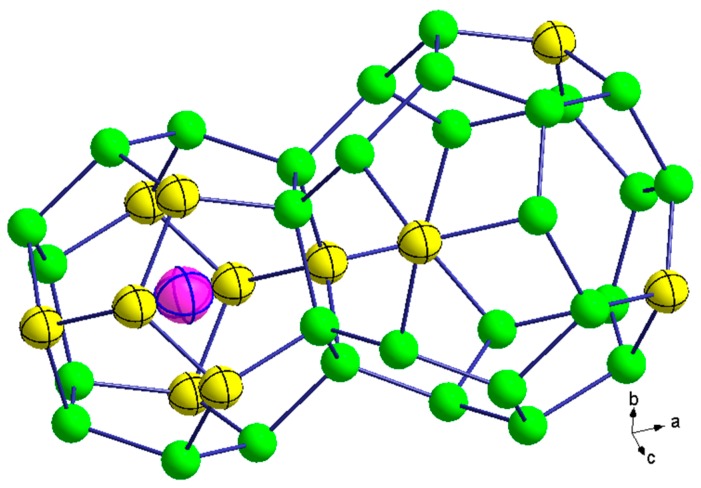
The dodecahedron (K,Ba) and hexakaidecahedron (empty) with thermal ellipsoids for all atoms corresponding to 95% probability for K_2.9(4)_Ba_13.1(2)_Ga_23.2(3)_Sn_112.7(5)_. Pink, yellow, and green spheres represent K/Ba (16*c*), Sn (32*e*), and Ga/Sn (8*a* and 96*g*), respectively.

**Figure 2:**
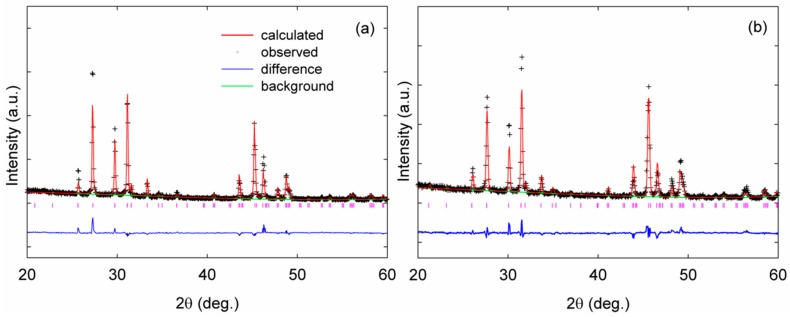
Powder X-ray diffraction (PXRD) data for (**a**) K_2.9(4)_Ba_13.1(2)_Ga_23.2(3)_Sn_112.7(5)_ and (**b**) K_7.1(2)_Ba_8.8(3)_Ga_25.1(4)_Sn_110.8(3)_, including profile fit, profile difference, profile residuals, and Bragg positions (purple ticks) from Rietveld refinement.

**Figure 3:**
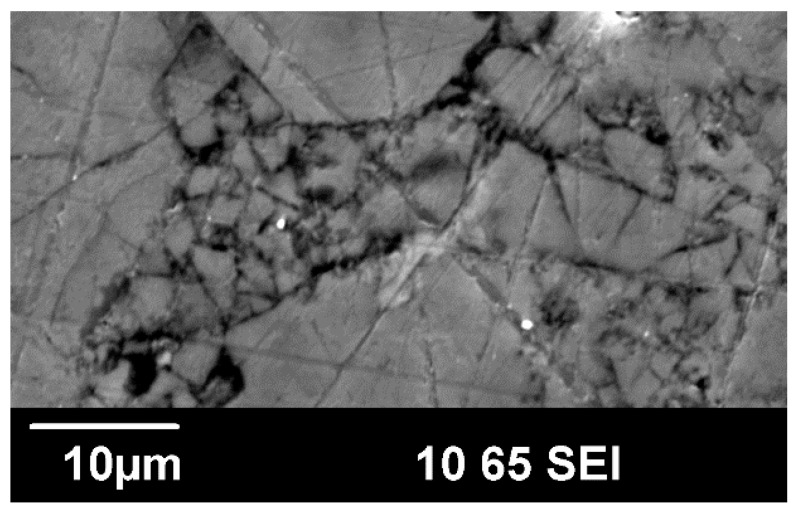
SEM image after the surface of the K_2.9(4)_Ba_13.1(2)_Ga_23.2(3)_Sn_112.7(5)_ specimen was cracked and roughed in order to reveal the grains.

The open-framework of clathrate-II (*Fd*$\bar{3}$*m*, No. 227-2) compounds comprise 136 tetrahedrally coordinated framework atoms per unit cell, located on crystallographic 96*g*, 32*e*, and 8*a* sites. The clathrate-II crystal structure is typically thought of as being built up of two types of polyhedral—(Ga,Sn)_20_ dodecahedra centered at 16*c* crystallographic sites and (Ga,Sn)_28_ hexakaidecahedra centered at 8*b* crystallographic sites (Figure 1) [3]. There are sixteen dodecahedra and eight hexakaidecahedra per conventional unit cell. As indicated by the residuals of the refinements shown in Table 1, with the fits shown in Figure 2, the PXRD refinement results show that each bulk polycrystalline specimen contains grains that are relatively compositionally homogenous after SPS processing, even if there were variations in the stoichiometries of the many single-crystals used to prepare the polycrystalline specimens. Table 2 shows one single-crystal refinement result as an example. This preparation method may therefore provide a unique approach in processing polycrystalline clathrate compositions that are similar to that of flux-grown single crystals for high temperature thermoelectric properties measurements, given the fact that such transport properties measurements have critical specimen size requirements that are often not easily obtained from the single-crystal growth of clathrates. A SEM image of K_2.9(4)_Ba_13.1(2)_Ga_23.2(3)_Sn_112.7(5)_ is shown in Figure 3 as an example of the grain morphology, and indicates grain sizes of 1 to 30 μm. As shown in Table 1, both K and Ba fully occupy the (Ga,Sn)_20_ dodecahedra, no alkali are present inside the hexakaidecahedra, and no vacancies are present on the framework. EDX analyses indicate compositions of K_7_Ba_9_Ga_26_Sn_110_ and K_3_Ba_13_Ga_23_Sn_113_ for K_7.1(2)_Ba_8.8(3)_Ga_25.1(4)_Sn_110.8(3)_ and K_2.9(4)_Ba_13.1(2)_Ga_23.2(3)_Sn_112.7(5)_, respectively, indicating relatively good agreement with the XRD refinement results. Our results are in agreement with that of Shäfer et al. [21]. The change in lattice parameters of our two specimens is very similar to that obtained by Shäfer et al. [22] on single crystals; the lattice parameters decrease linearly with increasing Ga content, as would be expected from the fact that Ga is much smaller than Sn on the framework, while K^+^ and Ba^2+^ are very similar in size [36]. On the other hand, our results differ from the experimental results of Koda et al. [19,20], in that those later two reports indicate “guest” species on the 8*b* crystallographic site. The difference in our processing approach and that reported in references 19 and 20 may result in a difference in stoichiometries.

An important factor contributing to the formation and occupancy of the two polyhedra in Sn clathrate-II materials is the relative size(s) of the constituent atoms. We note that our refinement results indicate that the framework is composed of a randomized distribution of Ga and Sn atoms on the 96*g* and 8*a* crystallographic sites; i.e., there is a tendency to avoid direct Ga–Ga connection. This minimizes the distortion of the polyhedra due to the different sizes of the Ga and Sn atoms, as has been noted by computational investigations of other Ga-containing Sn clathrate-II systems [37,38]. It is also apparent that the size of the guest atom affects the population of the specific polyhedra. In fact, the preferred location of the relatively small “guest” ions (relative to the size of the two polyhedral) is in the smaller dodecahedra, while Sn clathrate-II materials containing Cs or Rb guests (Cs_8_Ba_16_Ga_40_Sn_96_ and Rb_10_Ba_13_Ga_36_Sn_100_, for example) occupy the larger hexakaidecahedra [21]. Further evidence that K and Ba occupy only the dodecahedra comes from our recent first principles simulation studies of different Sn clathrate-II systems [37], indicating this to be an energetically more favorable arrangement than that of full occupancy of the polyhedra. In fact, comparing different Sn clathrate-II systems shows that the formation energy of K_2_Ba_14_Ga_30_Sn_106_ is 0.118 eV/atom lower than that of K_8_Ba_16_Ga_40_Sn_96_, while K_2_Ba_14_Ga_30_Sn_106_ was found to be 0.079 eV/atom more stable than Cs_8_Ba_16_Ga_40_Sn_96_, further indicating better energetic stability of Sn clathrate-II systems with the smaller polyhedra filled and the larger polyhedra empty [37].

**Figure 4:**
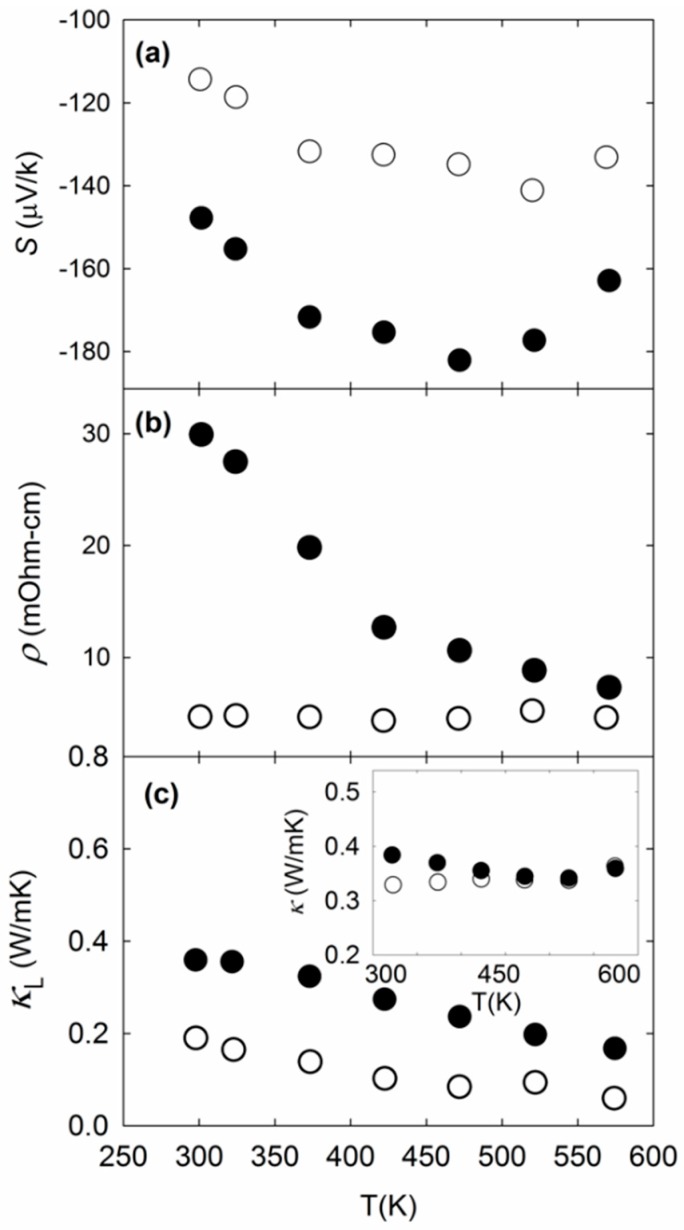
(**a**) *S*; (**b**) *ρ*; and (**c**) *κ*_L_ of K_2.9(4)_Ba_13.1(2)_Ga_23.2(3)_Sn_112.7(5)_ (empty circles) and K_7.1(2)_Ba_8.8(3)_Ga_25.1(4)_Sn_110.8(3)_ (filled circles). The *κ*_L_ values were estimated using the Wiedemann–Franz relation *κ*_L_ = *κ* − *κe*, where *κe* (= *L*_0_*T*/*ρ*) is the electronic contribution and the Lorenz number, *L*_0_, is taken to be 2.0 × 10^−8^ V^2^/K^2^. The inset to (**c**) shows the measured *κ* values of both specimens, with symbols as denoted previously.

Figure 4 shows *S*, *ρ*, and *κ*_L_ (the lattice contribution to *κ*) of the two specimens. The temperature-dependent *S* data indicates possible bipolar diffusion, as also observed by Koda et al. [19]. The resistivity trends somewhat with composition, K_2.9(4)_Ba_13.1(2)_Ga_23.2(3)_Sn_112.7(5)_ possessing lower *ρ* values in the measured temperature range as compared with K_7.1(2)_Ba_8.8(3)_Ga_25.1(4)_Sn_110.8(3)_, which shows more semiconducting-like behavior. This is expected, given the simple chemical analysis whereby each Ga acts as an acceptor for each donated electron from K^+^ and Ba^2+^. Similar behavior with composition was observed for other clathrate-I compositions [39,40]. Room temperature Hall measurements indicated carrier concentrations of 3.1 × 10^19^ cm^−3^ and 6.2 × 10^19^ cm^−3^ for K_7.1(2)_Ba_8.8(3)_Ga_25.1(4)_Sn_110.8(3)_ and K_2.9(4)_Ba_13.1(2)_Ga_23.2(3)_Sn_112.7(5)_, respectively, values that are in the same order-of-magnitude as that of previous reports on clathrate-II Sn compositions [18,19,20]. The effective mass is related to a material’s electronic structure. From our measured room temperature carrier concentration and *S* values, and assuming a simple parabolic band model [41], we estimated the effective mass m* of the two specimens to be 0.45*m*_e_ and 0.51*m*_e_ for K_7.1(2)_Ba_8.8(3)_Ga_25.1(4)_Sn_110.8(3)_ and K_2.9(4)_Ba_13.1(2)_Ga_23.2(3)_Sn_112.7(5)_, respectively. It was reported that m* for K_8_Ba_16_Ga_40_Sn_96_ calculated assuming a simple parabolic approximation of the energy bands led to smaller values as compared to experimentally-determined data [20]. The reason for this discrepancy may be because the energy bands near the Fermi level are not entirely parabolic and have a multi-valley character. The effective mass estimated from the density of states for K_8_Ba_16_Ga_40_Sn_96_ was reported to be 0.6*m_e_* [20], in general agreement with the experimentally estimated values for our clathrate compositions.

The carrier transport properties are expected to correlate with specific features in the electronic structure of both materials, especially around the Fermi level region. First principles calculations by Koda et al. [19] have shown that K^+^ and Ba^2+^ ions typically affect the conduction region, where dispersive bands due to coupling with the “guest” atoms are formed close to the Fermi level. The valence band region, however, is primarily due to the framework atoms. We note that the band structures for Sn_136_ and K_8_Ba_16_Sn_136_ are rather similar, with comparable semiconducting gaps (~0.7 eV) at the characteristic L-point. [19] For the mixed framework material K_8_Ba_16_Ga_40_Sn_96_, the reported calculations show that the band gap shifts to the characteristic R-point and is reduced to ~0.2 eV. This value is in the same range as our estimates for K_7.1(2)_Ba_8.8(3)_Ga_25.1(4)_Sn_110.8(3)_ (0.18 eV) and K_2.9(4)_Ba_13.1(2)_Ga_23.2(3)_Sn_112.7(5)_ (0.15 eV), experimentally determined from the equation *E*_g_ = 2*eT*_max_*S*_max_, where *E*_g_ is the band gap, *e* is the elementary charge, and *T*_max_ is the absolute temperature at the peak *S* value, *S*_max_ [42].

**Figure 5:**
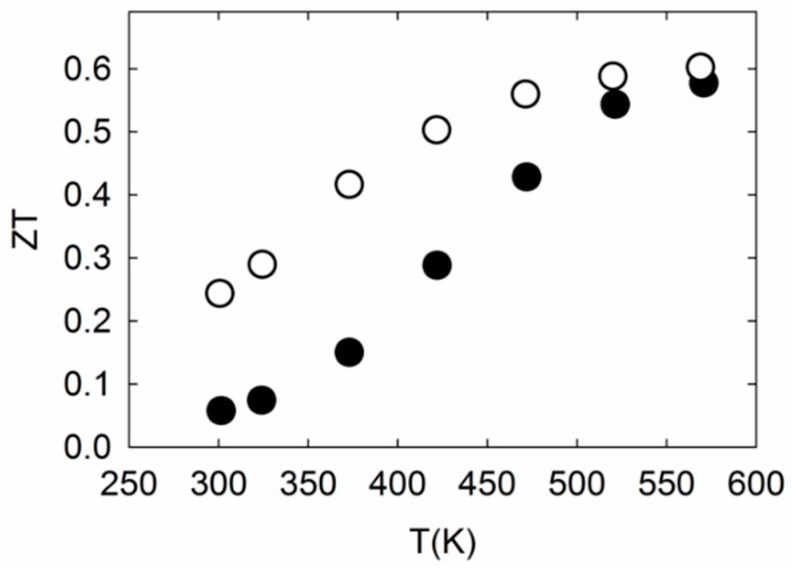
*ZT* of K_2.9(4)_Ba_13.1(2)_Ga_23.2(3)_Sn_112.7(5)_ (empty circles) and K_7.1(2)_Ba_8.8(3)_Ga_25.1(4)_Sn_110.8(3)_ (filled circles).

As shown in Figure 4c, *κ* is very low for these clathrates. The large unit cell and complex crystal structure of clathrate-II compounds contribute to the very low *κ*_L_, lower than most thermoelectric materials [43]. We note that the *κ*_L_ of both specimens are lower than that of previous reports on clathrate-II Sn compositions [18,19,20,21]. Mass fluctuation scattering between the filled and empty polyhedral cages, as well as between K and Ba inside the dodecahedra, contribute to the strong phonon scattering and subsequent low *κ*_L_ values for these compositions. This would not be the case for clathrates with fully filled and ordered ions inside the polyhedra. The “up-turn” in *κ* at ~500 K in both specimens may be due to bipolar thermal diffusion, as also observed in the *S* data. Similar power factor (*PF = S*^2^/*ρ*) and *κ* values above 500 K for both specimens resulted in similar values for the thermoelectric figure of merit, *ZT* = *S*^2^*T*/*ρκ*, with a slightly higher *ZT* value at 575 K corresponding to K_2.9(4)_Ba_13.1(2)_Ga_23.2(3)_Sn_112.7(5)_ (Figure 5). The plateauing of *ZT* at higher temperatures may suggest a limitation in further improving the thermoelectric properties of these Sn clathrate-II compounds by simple adjustment of their compositions.

## 4. Conclusions

Potassium- and barium-filled, gallium framework-substituted tin clathrate-II single crystals were synthesized in large quantities so that they may be ground to fine powder for densification, employing optimized SPS parameters to form dense bulk polycrystalline specimens for high temperature transport properties measurements. Our PXRD refinement results indicated a mixed occupancy in the (Ga,Sn)_20_ dodecahedra, vacant (Ga,Sn)_28_ hexakaidecahedra, and no vacancy on the framework. This type of preferential polyhedral occupancy can be related to the relative sizes of the constituent atoms. A plateau in the *ZT* values at higher temperatures was observed for both specimens, with the highest *ZT* value (0.6) achieved for K_2.9(4)_Ba_13.1(2)_Ga_23.2(3)_Sn_112.7(5)_ at 575 K.

## Figures and Tables

**Table 1:** Crystallographic details and selected bond distances for K_7.1(2)_Ba_8.8(3)_Ga_25.1(4)_Sn_110.8(3)_ and K_2.9(4)_Ba_13.1(2)_Ga_23.2(3)_Sn_112.7(5)_.

K_7.1(2)_Ba_8.8(3)_Ga_25.1(4)_Sn_110.8(3)_		K_2.9(4)_Ba_13.1(2)_Ga_23.2(3)_Sn_112.7(5)_
Space group, Z	*Fd*$\bar{3}$*m*, 1	
*a*, Å	17.0001 (7)	17.0182 (5)
*V*, Å^3^	4913.10 (6)	4928.80 (4)
Radiation	Graphite monochromated Cu Kα (1.5405 Å)	
T (K)	295	295
θ limits, deg.	5 to 30	5 to 30
R indices	0.1637, 0.0995 (wRp, Rp) ^a^	0.1261, 0.0992 (wRp, Rp) ^a^
Goodness-of-fit on F^2^	2.08	2.23
Ga_1_/Sn_1_ *x* and *y*	0.06791	0.06794
Ga_1_/Sn_1_ *z*	0.37267	0.37276
Sn_2_ *x*, *y*, and *z*	0.21816	0.21815
Ga_3_/Sn_3_ *x*, *y*, and *z*	0.125	0.125
*U*_iso_ for K/Ba	0.0146	0.0209
*U*_iso_ for Ga_1_/Sn_1_	0.0141	0.0145
*U*_iso_ for Sn_2_	0.0149	0.0142
*U*_iso_ for Ga3/Sn_3_	0.0121	0.0286
K/Ba—Ga_1_/Sn_1_, Å	3.90693 (12)	3.91165 (8)
K/Ba—Sn_2_, Å	3.78691 (15)	3.79083 (11)
K/Ba—Ga_3_/Sn_3_, Å	3.68062 (11)	3.68454 (8)
Ga_1_/Sn_1_—Sn_2_, Å	2.76613 (10)	2.77084 (7)
Sn_2_—Ga_3_/Sn_3_, Å	2.74310 (8)	2.74572 (6)

^a^ wR_p_ = ((Σ*w*(I_o_ − I_c_)^2^/Σ*w*I_o_^2^)^1/2^ and R_p_ = Σ|I_o_ − I_c_|/ΣI_o_; Equivalent position: (+*x*, +*y*, +*z*), (+*x* + 1/4, +*y* + 1/4, −*z*), (−*z* + 1/4, +*x* + 1/2, −*y* + 3/4), (−*x* + 3/4, +*y* + 1/2, −*z* + 1/4).

**Table 2:** Single-crystal refinement result for a crystal used in processing the K_7.1(2)_Ba_8.8(3)_Ga_25.1(4)_Sn_110.8(3)_ polycrystalline specimen.

Stoichiometry	K_6.9(4)_Ba_8.9(5)_Ga_23.5(2)_Sn_112.3(3)_
Space group, Z	*Fd*$\bar{3}$*m*, 1
*a*, Å	17.0006 (3)
*V*, Å^3^	4917.08 (2)
Radiation	Cu Kα, INCOATEC Imus micro-focus source (*λ* = 1.54178 Å)
T (K)	100
Absorption coefficient (mm^−1^)	10.615
θ limits, degree	6.07 to 72
No. Unique data with $F_{o}^{2}$	6310/210 [R(int) = 0.0386]
No. of unique data with $F_{o}^{2}$ > 2σ ($F_{o}^{2}$)	212
R indices	0.0508, 0.0370 (wR2, R1) ^a^
Goodness-of-fit on F^2^	2.994
Max. and Min. residual e- density(e/Å^3^)	0.260/−0.259

^a^ wR2 ($F_{o}^{2}$ > 0) and R1 ($F_{o}^{2}$ > 2σ($F_{o}^{2}$)).
